# An unusual split fracture of the fourth and fifth metacarpal heads in a young adolescent: a case report

**DOI:** 10.1097/RC9.0000000000000320

**Published:** 2026-03-02

**Authors:** Anthony El Dada, Wendy Ghanem, Rayan Elias, Rima Osman, Hady Ezzeddine, Ramzi Moucharafieh

**Affiliations:** a Department of Orthopedics and Traumatology, Faculty of Medicine, University of Balamand, Beirut, Lebanon; b Department of Orthopedics and Traumatology, Clemenceau Medical Center, Beirut, Lebanon; c European University Cyprus, Engomi, Cyprus

**Keywords:** case report, headless compression screws, head-split fracture, intra-articular, metacarpal head fractures, open reduction

## Abstract

**Introduction and Importance:**

Metacarpal head fractures are rare intra-articular injuries categorized into 10 types by McElfresh and Dobyns. This report discusses the first reported case of simultaneous coronal head-split fractures of the fourth and fifth metacarpals, the unusual mechanism of injury, and our surgical management of this never-seen type of fracture.

**Case Presentation:**

We describe the case of a coronal head split fracture of the fourth and fifth metacarpals in an 18-year-old male who presented with severe pain in the knuckles following a punch. Radiographs of the left hand showed intra-articular fractures of the heads of his fourth and fifth metacarpals step-off of 3 mm. Fixation was performed using two crossed 1.2-mm headless compression screws, each inserted dorsal to palmar to restore articular surface integrity and congruency. A full active range of motion was achieved at 8 weeks postoperatively without rotational deformity.

**Clinical Discussion:**

We present our approach to patient management, comparing it to the existing literature. Given the articular step-off and the patient’s young age, open reduction and internal fixation with headless compression screws was performed. To the best of our knowledge, this is the first documented case of surgical management for this specific fracture pattern, highlighting our successful approach in achieving anatomical reduction and stable fixation, which facilitated early mobilization and a favorable outcome.

**Conclusion:**

This report highlights a viable surgical approach and major take-away points for this rare intra-articular injury, namely fracture pattern recognition, adherence to surgical principles, and achieving appropriate anatomical reduction and stable fixation to promote faster and successful recovery.

## Introduction

Metacarpal fractures account for approximately 10% of all adult fractures[1]. These injuries can occur at the metacarpal base, shaft, neck, or head. While fifth metacarpal neck (boxer’s) fractures are common, intra-articular head fractures are particularly rare^[^2,3^]^. In 1997, McElfresh and Dobyns[4] categorized intra-articular metacarpal head fractures into 10 types: epiphyseal, osteochondral, collateral ligament avulsion, coronal, oblique, and horizontal, boxer-type with intra-articular split, comminuted, fractures with loss of substance, and occult fractures leading to avascular necrosis. However, simultaneous coronal head-split patterns are not traditionally described.

HIGHLIGHTS

- First reported case of simultaneous coronal head-split fractures of fourth and fifth metacarpals.
- Injury occurred after punch in flexion, differing from usual axial load mechanism.
- Open reduction and internal fixation with headless screws allowed anatomical reduction and early mobilization.
- Full functional recovery achieved by 8 weeks without complications.
- Highlights importance of stable fixation to prevent arthritis and avascular necrosis.

We present an 18-year-old male with a coronal head-split fracture of the fourth and fifth metacarpals. The fractures were intra-articular, sparing the metacarpal necks following a punch (in flexion), differing from the usual axial load mechanism. To the best of our knowledge, no similar cases have been documented. We additionally describe our management and compare it to other approaches reported in the literature for different types of intra-articular metacarpal head fractures (Table 1). This case report has been reported in line with the SCARE checklist[5].

**Table 1:** Reported cases of metacarpal intra-articular head fractures and their different management approaches

Author and year	Metacarpal head involved	Fracture type	Management	Follow-up duration (months)	Follow-up findings
Mousafeiris *et al*[3] (2021)	Fourth	Transverse (horizontal)	Kirschner wires	9	Partial avascular necrosis of the metacarpal head, normal function.
Pandya *et al*[6] (2020)	Fifth	Oblique (non-complete)	Conservative	1	No increased laxity of the collateral ligament, normal function of the hand.
Seaton *et al*[7] (2023)	Fifth	Comminuted	External fixation	4	Minimal stiffness
Kato *et al*[8] (2020)	Second	Coronal and epiphyseal	Cortical screw fixation	12	70^o^ of flexion, 10^o^ of extension at the metacarpophalangeal joint, compared to 80^o^ in the contralateral hand. No signs of avascular necrosis.

## Case presentation

### Patient information

An 18-year-old male, a non-smoker with celiac disease, was admitted to the emergency department for pain and swelling of the left hand following a punch to the wall. The patient had no relevant comorbidities or family history impacting bone or joint health. He also had no relevant past surgical history or food or drug allergies. The patient arrived at the emergency department complaining of severe pain in the knuckles.

### Clinical findings

Physical examination of the left hand showed severe tenderness and swelling over his fourth and fifth knuckles. There was no soft tissue loss or laceration noted, and the neurovascular status of the left upper limb was intact.

### Diagnostic assessment & interpretation

**Figure 1.**
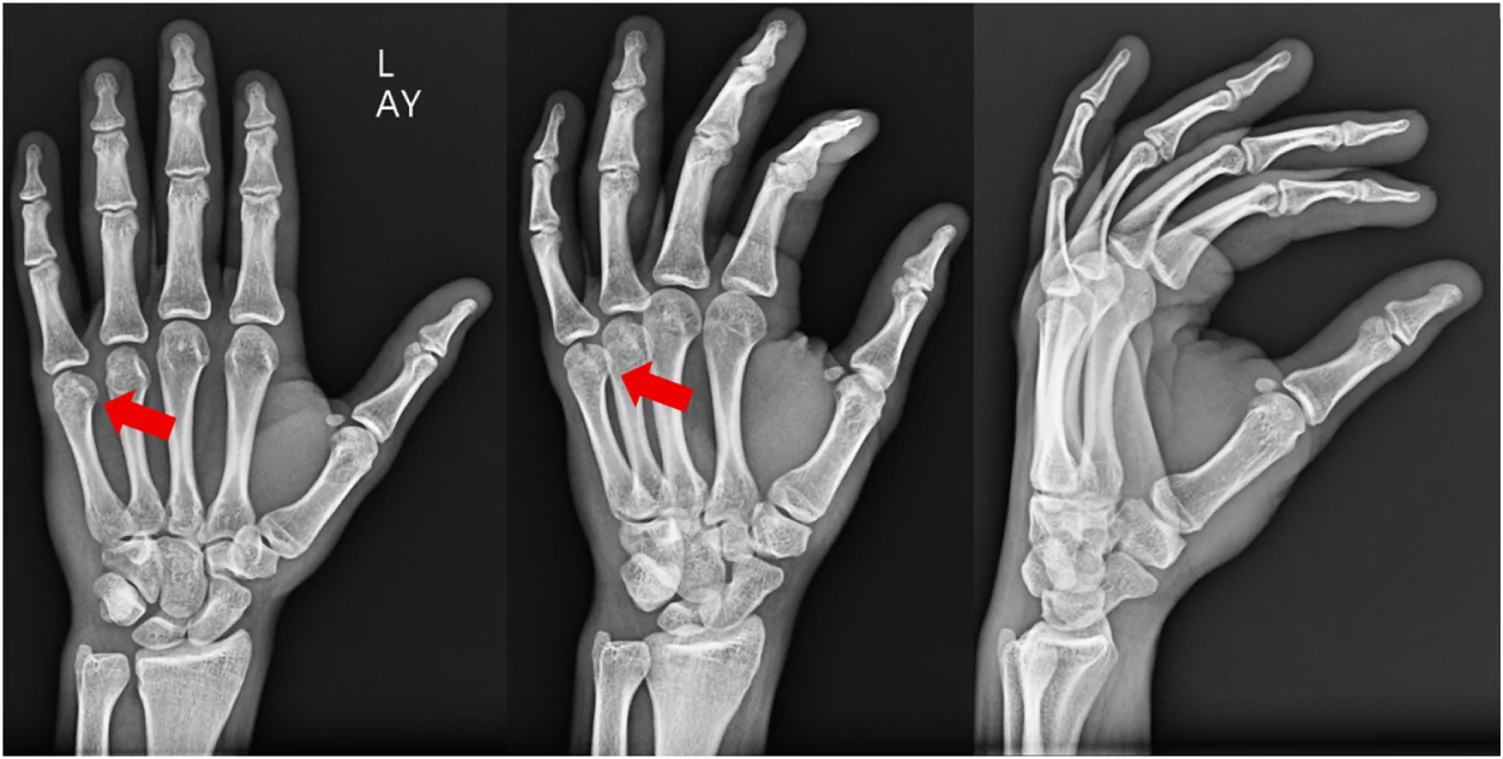
Posteroanterior, oblique, and lateral radiographs of the left hand showing intra-articular head split fractures of the fourth and fifth metacarpals.

Radiographs of the left hand showed intra-articular fractures of the heads of his fourth and fifth metacarpals step-off of 3 mm (Fig. 1). Accordingly, the patient qualified for open reduction internal fixation.

### Intervention

The patient followed standard preoperative patient optimization and was admitted for open reduction and internal fixation (ORIF). Under a left upper limb supraclavicular nerve block, using two separate dorso ulnar incisions centered over the fourth and fifth metacarpal heads, the metacarpophalangeal joints were exposed through the extensor apparatus and joint capsules on the ulnar side.

**Figure 2.**
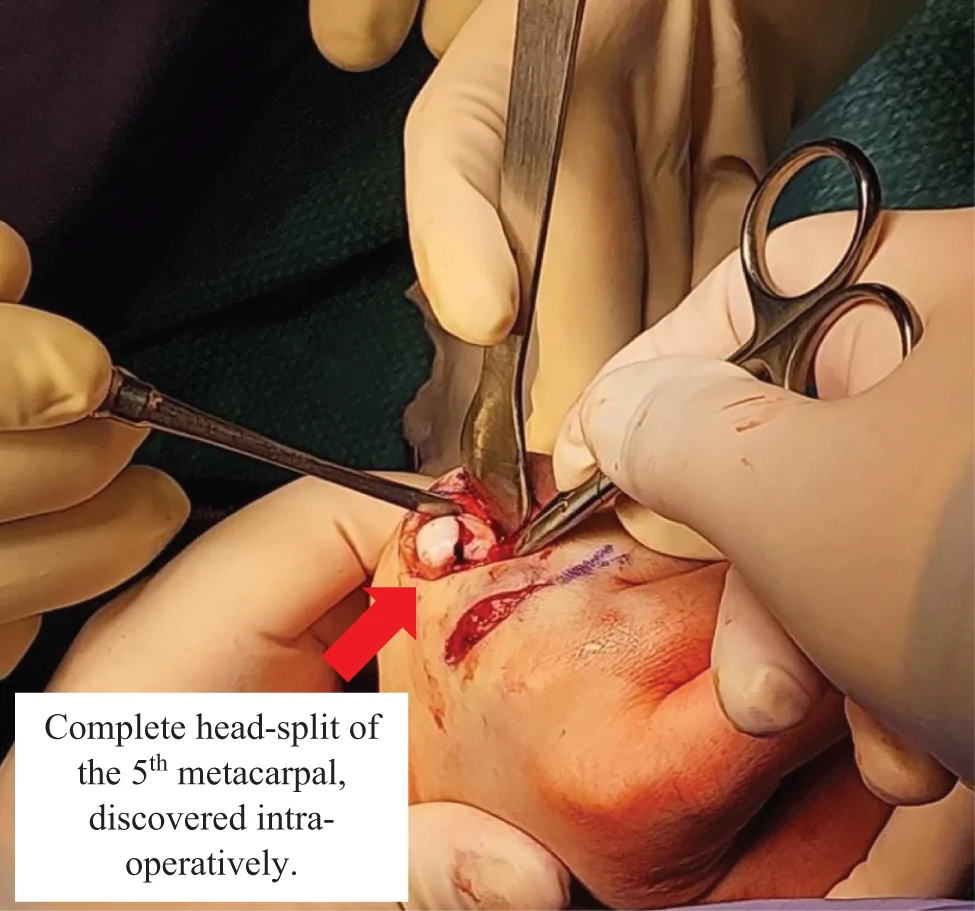
Intraoperative picture showing complete head-split of the fifth metacarpal head.

**Figure 3.**
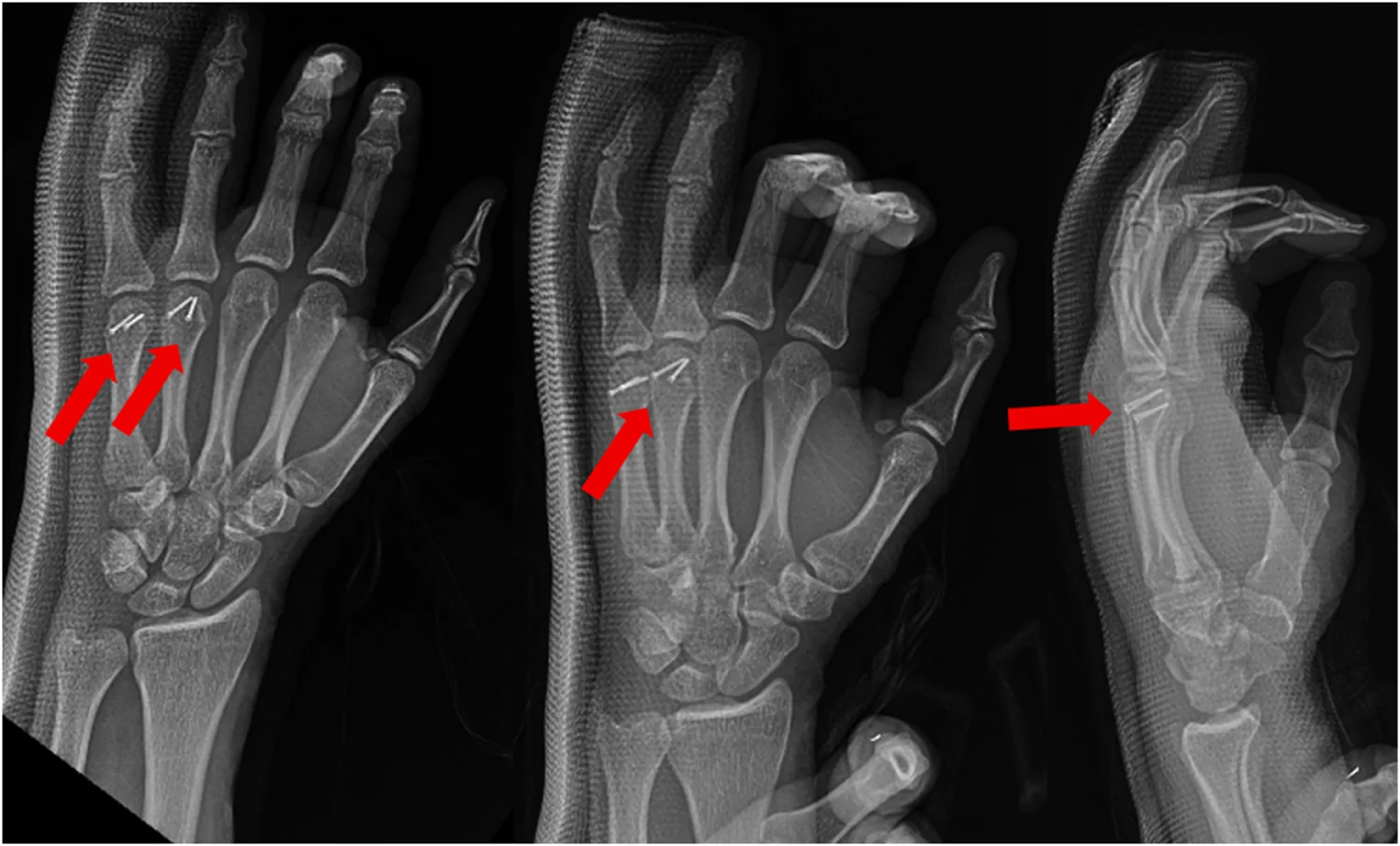
Posteroanterior, oblique, and lateral radiographs of the left hand showing reduction of the fourth and fifth metacarpal fractures with good bony alignment.

Fractures were exposed, revealing the coronal head split fractures (Fig. 2). Although the fracture pattern was novel and unexpected, we did not deviate from our initial management plan and adhered to the principles of intra-articular fracture management. The fractures were reduced and temporarily stabilized with Kirschner wires, with confirmation under fluoroscopy under multiple views (PA, oblique). Definitive fixation was achieved using two crossed 1.2-mm headless compression screws inserted dorsal to palmar, with subsequent fluoroscopic confirmation. Closure in layers was performed (capsule, extensor apparatus, fascia, and subcutaneous tissue), and an ulnar gutter splint and arm sling were placed for immobilization. Postoperative radiographs confirmed good alignment of the reduced fourth and fifth metacarpal bones (Fig. 3).

### Patient follow-up and outcomes

**Figure 4.**
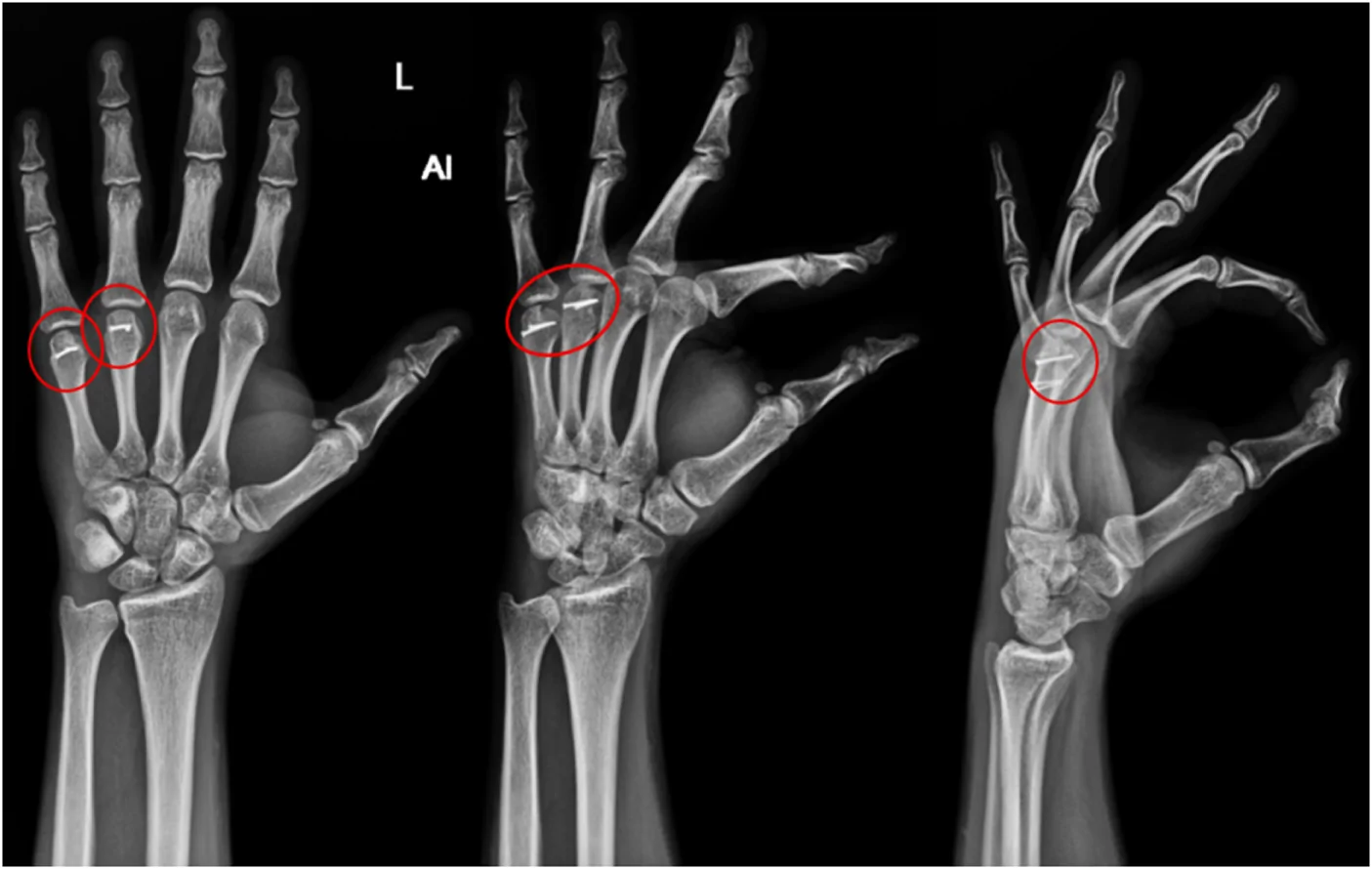
Posteroanterior, oblique, and lateral radiographs of the left-hand showing reduction of the fourth and fifth metacarpal fractures with good bony alignment at 2 months post-op.

**Figure 5.**
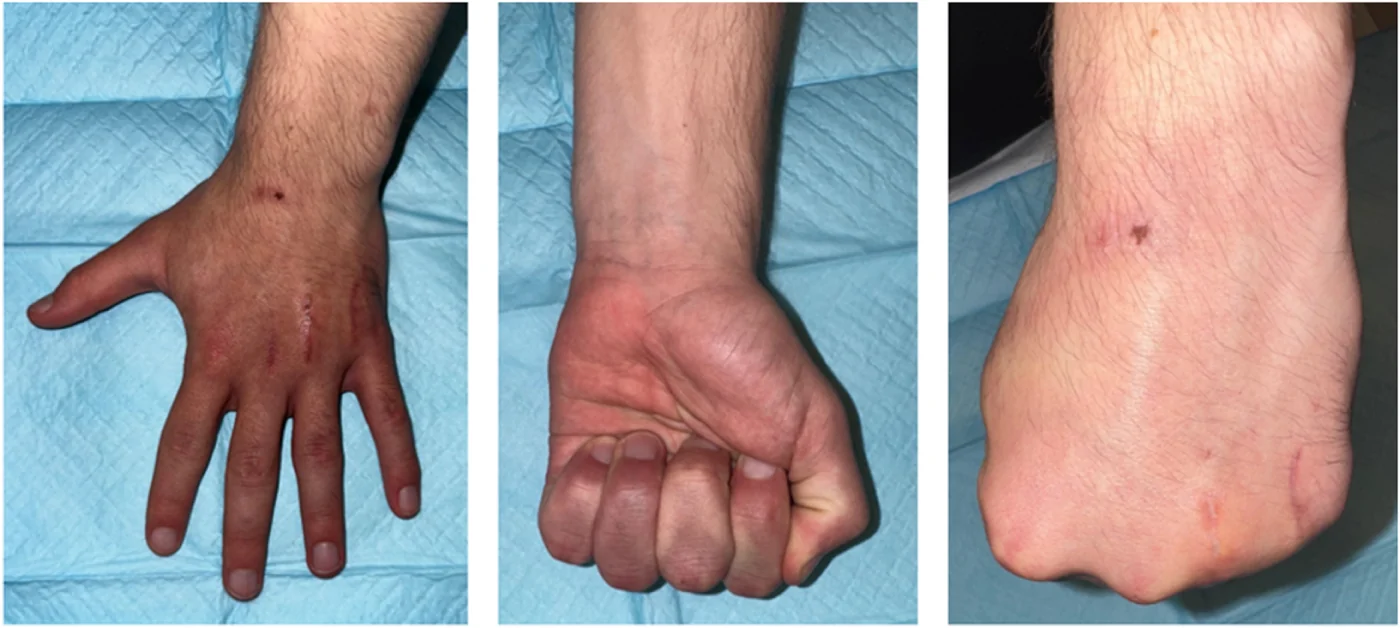
Physical exam at 2 months post-op showing full range of motion and no rotational deformity of fourth and fifth digits.

The patient’s splint was removed at 10 days post-op, and early protected active, assisted active, and gentle passive range of motion was started. Follow-up X-rays at 8 weeks postoperatively showed the absence of any articular step-off, good reduction, and good alignment (Fig. 4). Also, full active range of motion (metacarpophalangeal [MCP] flexion 0°–90°) was achieved without rotational deformity, comparable to the contralateral hand (Fig. 5). Contact sports were prohibited for 3 months. Follow-up imaging and clinical evaluation were recommended at 6 and 12 months postoperatively to assess joint congruency and avascular necrosis risk.

### Complications and adverse events

No intraoperative or postoperative complications occurred. Blood loss was minimal. No infection, hardware migration, or adverse drug reactions were observed. Hospital discharge occurred within 24 hours.

## Discussion

Horizontal metacarpal head split fractures, a rare type of intra-articular metacarpal head fracture, are usually associated with axial compression of the extended digit[3], contrary to our patient who sustained his coronal split fracture following a punch (in flexion of the MCP joint), which is commonly the mechanism associated with a boxer’s fracture[9]. In this case, we hypothesize that the punch in flexion created an ulnar-directed load transfer that caused simultaneous shearing across both metacarpal heads. Another hypothesis is that this patient’s celiac disease, known to increase fracture risk overall[10], predisposed him to an abnormal fracture pattern.

Metacarpal head fractures are usually diagnosed through posteroanterior and oblique radiographs, but the fracture pattern is not always apparent. Similar to other types of intra-articular fractures, a three-dimensional CT scan can aid in confirming the diagnosis, determining the fracture pattern, and planning the fixation^[^11,12^]^. In our case, however, the fracture was evident on standard X-rays, and a CT scan was not necessary.

Different approaches for intra-articular metacarpal head fracture management were described, including conservative treatment, Kirschner wire fixation, external fixation, and ORIF with headless compression screws^[^3,6–8^]^. ORIF with headless compression screws has been gaining popularity over conventional fixation methods, seeing that they allow early range of motion and rehabilitation^[^13,14^]^. Due to the patient’s young age and the presence of an articular step-off, the decision for ORIF with headless screws was taken. Anatomical reduction is necessary to avoid the risk of MCP joint osteoarthritis, avascular necrosis, and chronic pain. ORIF can be challenging in some cases, and even when successful, the articular surface may experience significant changes, leading to joint stiffness, osteoarthritis, and chronic pain[15]. Upon literature review, there were no cases reporting a coronal metacarpal head split fracture, let alone its management. However, similarly to any articular fracture, current studies favor ORIF over conservative treatment for metacarpal head fracture, allowing accurate reduction, stable fixation, and early range of motion[16].

Our patient did not have any preoperative risk factors (smoking, alcohol abuse, corticosteroid use…) that predisposed him to avascular necrosis. Wright and Dell conducted a metacarpal vascular anatomy study where they identified the absence of large nutrient vessels in 35% of specimens, placing the distal metacarpal epiphyses at risk of avascular necrosis development and making these metacarpal heads solely dependent on small circumferential pericapsular arterioles[17]. Any insult to these small pericapsular vessels might jeopardize the vascularity of the metacarpal head, leading to avascular necrosis. In a traumatic setting, avascular necrosis of the metacarpal head can be the result of an occult fracture or secondary to a traumatic effusion^[^18–20^]^. In addition, displacement of fracture fragments, comminution, involvement of second and third metacarpal heads, and soft tissue loss were also recognized as possible risk factors for metacarpal head avascular necrosis[4]. On that note, and precluding that the risk of avascular necrosis is present and uncertain, the management of a metacarpal head fracture should aim for an anatomical reduction and atraumatic surgery, achieving joint congruency and maintenance of a stable yet flexible periarticular envelope, which, in turn, might be beneficial to the revascularization of the bone fragments.

The main lesson from this case is the importance of recognizing fracture patterns. While standard radiographs may be enough for diagnosis, careful intraoperative attention to the fracture morphology is critical in guiding management. Anatomical reduction and rigid fixation with headless compression screws were essential to restore joint congruity, allow early mobilization, and minimize the risks of stiffness, arthritis, and avascular necrosis.

Limitations of this report include its single-patient nature, absence of CT imaging for preoperative planning, lack of a formal long-term functional scoring (e.g., DASH), and relatively short follow-up duration. Nonetheless, intraoperative findings confirmed the coronal head-split pattern, and the patient’s quick recovery supports the role of stable fixation and early motion in such rare injuries.

In summary, this case contributes to the sparse literature by describing, to the best of our knowledge, the first reported instance of simultaneous coronal head-split fractures of the fourth and fifth metacarpals. Early recognition, anatomical reduction, and stable internal fixation allowed for a full functional recovery. Hand surgeons should be aware of this rare pattern and apply principles of articular fracture management to optimize long-term outcomes.

## Conclusion

Metacarpal head split fracture is a rare type of intra-articular metacarpal head fracture. The literature offers different strategies to approach metacarpal head fractures, ranging from conservative to surgical. However, there were no cases reported of a coronal split fracture of a metacarpal head in the literature. Therefore, we presented a case of an 18-year-old male found to have a left fourth and fifth metacarpal head split fractures treated with ORIF using headless compression screws.

Our report aims to highlight important takeaway lessons. First, fracture pattern recognition is necessary intraoperatively as radiographs may not immediately reveal the true pattern of a fracture. Second, adherence to surgical principles is a must. Even in very rare fracture patterns, the principles of treating intra-articular fractures remain the same. Finally, anatomical reduction and stable fixation should always be the end-goal; our approach promotes a faster and more favorable return to function, making it a viable strategy for managing this type of injury.

## Data Availability

The data that support the findings of this study are available from the corresponding author upon reasonable request.
